# Evaluation of Hematological Parameters in Partial Exchange and Packed Cell Transfusion in Treatment of Severe Anemia in Pregnancy

**DOI:** 10.1155/2012/608658

**Published:** 2012-05-28

**Authors:** Sudha Salhan, Vrijesh Tripathi, Rajvir Singh, Harsha S. Gaikwad

**Affiliations:** ^1^Department of Obstetrics & Gynecology, Hamdard Institute of Medical Sciences & Research, New Delhi 110062, India; ^2^The Department of Mathematics & Statistics, The University of the West Indies, St. Augustine Campus, Trinidad and Tobago; ^3^Medical Research Centre and Cardiology, CCS Department, Hamad Medical Corporation (HMC), Doha, Qatar; ^4^Department of Obstetrics & Gynecology, Vardhman Mahavir Medical College & Safdarjung Hospital, New Delhi 110029, India

## Abstract

*Objectives*. Anemia is a major public health problem throughout the world which assumes prominence in pregnant mothers. Patients with severe anemia continue to present themselves at term or in labor. This study was conducted to compare the improvements in hematological parameters of patients receiving partial exchange blood transfusion and transfusion of packed cells without exchange. *Methods*. One hundred and twenty-five severely anemic antenatal mothers were admitted from outpatient service. Partial exchange transfusion was given to sixty-six patients while fifty-nine received transfusion of packed cells with frusemide cover. *Results*. The two groups were comparable in terms of age, height, weight, religion, diet, education, occupation of self and husband, and income. Hemoglobin level in Group 1 was comparatively less than Group 2 at prelevel (5.2 ± 1.5 versus 6.6 ± 2.3, *P* = 0.001) and postlevel (7.2 ± 1.5 versus 8.6 ± 1.8, *P* = 0.001), respectively, but there was no significant difference between the two modes of transfusion (2.09 ± 1.6 versus 2.01 ± 1.5, *P* = 0.78). *Conclusion*. The study produced an equally significant improvement in hematological parameters in partial exchange and packed cell transfusion. Platelet counts were significantly less in partial exchange as compared with packed cell transfusion.

## 1. Introduction

Anemia has an estimated global prevalence of 42% among pregnant women. It is estimated that anemia causes more than 115000 maternal and 591000 perinatal deaths globally per year [1]. The baseline measure of hemoglobin concentration that categorizes anemia is less than 7.0 g/dL for severe anemia, 7.0–9.9 g/dL for moderate anemia, and 10.0–11.9 g/dL for mild anemia [2]. The estimated prevalence of anemia among pregnant women in India is higher than the global prevalence at 49.7% [3]. According to survey data, 84.9% of pregnant women are anemic with 60.1% having moderate anemia and 13.1% having severe anemia [4]. A study recorded that the prevalence of anemia in pregnant women and children among slum dwellers in Delhi was as high as 80.6% and 93.3%, respectively [5]. Anemia has been identified as a public health problem yet the problem is accentuated when pregnant women present themselves with severe anemia late in labor.

Various studies have assessed the association between severe anemia and maternal mortality. Brabin et al. in a review used cross-sectional, longitudinal, and case-control studies and found a strong association between severe anemia and maternal mortality. However, the association was not as strong for mild or moderate anemia [6]. Harrison found that both maternal and fetal mortality rise sharply in cases of severe anemia. The causes for maternal death were due to anemic heart failure, fulminating bacterial infection, and shock from even a small loss of blood at delivery or abortion [7]. Severe anemia was associated with a fourfold increase in risk of death in a study by Fullerton and Turner [8].

Women often become anemic during pregnancy because of the increase in demand for iron and other vitamins in the body. It is estimated that the blood volume increases approximately 50 per cent during pregnancy, although the plasma amount is disproportionately greater. This causes dilution of the blood, making the hemoglobin concentration fall, with hemoglobin concentration at its lowest between weeks 25 and 30. Despite anemia prevention programs and improved health services at all levels of health care in India, a fairly large number of patients still present with severe anemia in the third trimester and in labor. One of the motives of improving anemic status is to enable the patient to withstand the stress of labor. However, when patients present with severe anemia so late in pregnancy, there is no time left to allow correction of anemia by iron supplementation. In these patients, blood transfusion becomes the treatment of choice as it brings about an immediate improvement in the anemic status.

Blood transfusion can be given in three ways: packed cell transfusion without exchange, total exchange blood transfusion, and partial exchange blood transfusion without exchange. Partial exchange blood transfusion has evolved as a compromise between blood transfusion without exchange and total exchange transfusion. To avoid circulatory overload, it is necessary to increase the hemoglobin and red cell volume without raising the plasma volume and total blood volume [7]. A severely anemic antenatal mother has an expanded plasma volume with low Hb and PCV. Withdrawal of 350 mL of blood decreases the cardiac load. Since the sediment part of this withdrawn blood, which is rich in red cells, is retransfused, there is minimal loss of red cells and a significant reduction in plasma volume after the withdrawal.

Results from a study done by Philpott et al. in 1966 recommended exchange transfusion since it did not pose any increase in risk factors [9]. Singh et al. concluded that mortality was significantly low (14.8%) in severely anemic antenatal mothers in patients receiving partial exchange blood transfusion as compared to the mortality in patients of CHF (23%). Their study also concluded that partial exchange and whole blood transfusion produce an improvement in the oxygen carrying capacity of blood. The amount of extra red cells introduced in circulation is same in both the procedures (two units of whole blood). Hence, the improvement in Hb and PCV is comparable [10]. Harrison has also recommended the use of parenteral ethacrynic acid to prevent acute pulmonary edema, producing acute diuresis and a reduction of plasma volume in direct blood transfusion [11]. Our study was conducted to compare the effect of partial exchange blood transfusion and transfusion of packed cells with frusemide cover on hematological parameters in antenatal mothers with severe anemia.

## 2. Methodology

This was a comparative retrospective study conducted in the Department of Obstetrics and Gynaecology of Safdarjung Hospital, New Delhi, India, during the period June 2009 and January 2011. One hundred and twenty-five severely anemic antenatal mothers were recruited from the outpatient service and Emergency Department. Sixty-six patients with severe anemia who presented themselves in one of the units of the Department were administered partial exchange. In the other units there were fifty-nine patients with severe anemia which were given transfusion of packed cells with frusemide cover during the same time period. All parameters were recorded from patient record files while the patient was admitted in the hospital wards.

The data were divided into two groups. Group 1 comprised of women who received partial exchange blood transfusion and Group 2 comprised of women who received transfusion of packed cells with frusemide cover. The baseline investigations were recorded on a standard Performa. A detailed history of these patients was recorded including thorough general and complete physical examination of the patient. A detailed obstetric examination of patients was also carried out including assessment of the fetal well-being.

Of the sixty-six women in Group 1, 36 had dimorphic anemia, 25 had microcytic hypochromic anemia (iron-deficiency anemia), and 5 had normocytic anemia. Of the 59 women in Group 2, 32 had dimorphic anemia, 20 had microcytic hypochromic anemia (iron-deficiency anemia), and 7 had haemoglobinopathy. All patients had gross pallor, were anxious looking, slightly irritable and had severe degree edema in their feet. 55 had tachypnea and 56 had generalized anasarca.

All women were admitted to special cubicles for sick patients in propped-up position. Oxygen was started to those with breathlessness, frusemide was administered to those with overt signs of congestive heart failure, and physician referral was sought. Deworming was done in all patients and a high protein diet was advised. Peripheral smear was taken before transfusion. Hematological investigations were carried out to know the severity of anemia. The patient's temperature, pulse, respiratory rate, and blood pressure was checked at the beginning of the transfusion, 15 minutes following the start of the transfusion and again at the end of the unit. Patients were closely monitored for vitals and transfusion reactions for up to 24 hours after transfusion. In Group 1, two units were transfused at a time one after the other. In Group 2, one unit was given at a time on a single day. The volume of blood transfused was 250 mL of packed cells with the venesection size of 18 gauge cannula.

Pre- and post-transfusion changes were recorded through a complete blood count (CBC), liver and kidney function tests. The post-tests were conducted after 48 hours of blood transfusion. Ethical clearance was taken from Ethics Committee of the Hospital to publish the study.

## 3. Statistical Analysis

Descriptive statistics in the form of means and standard deviations for interval variables and frequency and percentages for categorical variables were calculated. To see statistical significance within variables for before and after, parametric and nonparametric tests such as Paired *t*-test and Wilcoxon Sign Rank test, were used for interval variables and McNemar's tests for categorical variables. Mann Whitney *U* test and Independent Student *t*-test were used for pre- and post-differences as well as between the groups for interval variables wherever applicable. *P* value of less than 0.05 (two tailed) has been considered for statistical significance level. SPSS 12.0 Statistical Package was used for the analysis.

## 4. Results

The study included one hundred and twenty-five antenatal mothers. Out of them sixty-six were given partial exchange blood transfusion (Group 1) whereas fifty-nine were given packed cell transfusion with frusemide cover (Group 2). Demographic details were recorded and are presented in Table 1. The average age of patients was 25 years and most patients were Hindu (94%) and nonsmokers (98%). 12% of husbands and 33% of patients were illiterate. 63% husbands were unskilled and 92% were nonworking housewives. There were no differences in subject's education, occupation, diet, and birth interval between the groups. Almost 50% of the total women were vegetarians. Women with parity of less than 2 were more in Group 2 than in Group 1, indicating that there were more women with higher parity in Group 1. The difference was statistically significant.

Height, weight, pulse rate, systolic and diastolic blood pressure, and respiratory rate were similar between the groups (Table 2). There was also no difference at temperature, icterus, cyanosis antenatal checkup frequencies, and general conditions of the subjects between the groups. None of the women reported any past disease except for one who had epilepsy. One pregnancy had two fetuses. Antenatal care (54.5% versus 75.9%, *P* = 0.02) and haematinics (52.1% versus 93.9%, *P* = 0.001) were taken more in Group 2 whereas edema (56.3% versus 31.6%, *P* = 0.006) and pallor (89.2% versus 74.1%, *P* = 0.03) were more in Group 1. Table 1 also shows that normal chest (82.8% versus 98.2, *P* = 0.005), normal cvs (80.6% versus 96.4%, *P* = 0.01), and fundal height (wks) (30.2 ± 6 versus 36.9 ± 9, *P* = 0.001) were more in Group 2 whereas other parameters included in Table 2 were comparable in both the groups except that there were more preterm babies (45.5% versus 14.3%, *P* = 0.009) in Group 1. 3 women died in Group 1. There were 4 reported cases of induced labor (2 oxytocin and 2 Prostaglandins).

Table 3 presents means and standard deviations of pre- and post-levels for CBC, liver and kidney functions showing Hb before (5 ± 1.6 versus 6.6 ± 2.3, *P* = 0.001) and after (7.2 ± 1.5 versus 8.6 ± 1.8, *P* = 0.001) higher in Group 2 than in Group 1. Similar trends were seen for pre- and post-platelet and pre- and post-bilirubin levels. Other parameters were not significant between the groups.

Table 4 shows the differences for pre- and post-levels of given hematological parameters. The table revealed that mean ranks were similar between the groups except platelet count which was less in Group 1 (mean rank 49.3 versus 61.8, *P* = 0.04) than in Group 2.

## 5. Discussion

There were sixty-six women with anemia in Group 1 and fifty-nine women in Group 2. The two groups were comparable in terms of age, height, weight, religion, diet, education, income, and occupation of self and husband. However, there were more nulliparous women in Group 2 than in Group 1. There were more women with parity more than 3, in Group 1 than in Group 2. The difference was statistically significant. A review of studies done in Malawi records that three studies found prevalence of severe anemia to be higher in primipara than in multipara adolescents, but the differences were not statistically significant [12]. Our study results did not corroborate with this.

According to a Lancet review article, vitamin B12 deficiency is associated with lactovegetarianism in India and the scarcity of meat products in many South-Asian diets [1]. However, our study did not show any baseline difference between the two groups in terms of their diets. Majority of women in Group 1 were severely anemic as compared with 64% in Group 2. 52% patients were taking haematinics in Group 1 as against 94% in Group 2. The difference between the two groups was statistically significant. However, the benefits of this supplementation or the regularity of the administration of this supplementation were not assessed in the present study, and therefore a direct relation between severely anemic and not taking of haematinics was not established. An important observation in the meta-analysis of Sloan, Jordan, and Winikoff was that though iron supplementation increased initial hemoglobin level, the extent of the effects is limited [13].

Edema and pallor were statistically significant in Group 1 as compared with Group 2. The presence of pallor is clinically indicative of severe anemia [14]. Our study corroborated with the results from this study. Majority of women had normal chest, CVS and abdomen at the time of admission. The onset of labor is statistically significant due to higher number of caesarian sections recorded in Group 2. However, there was a lot of missing data for Group 1, and therefore the finding was not assessed. Fundal height was significantly higher in Group 2 at 35.9 weeks than in Group 1 at 30.1 weeks.

In the present study, data regarding outcome of pregnancy and mother and child's health at discharge was available only in relation to 26 women in Group 1 and 23 women in Group 2. Group 1 had 7 still births or macerated IUD while there were 4 such in Group 2. There were more caesarian sections in Group 2 than in Group 1. Data regarding condition of baby at discharge showed that there were 13 healthy babies in Group 1 while 23 babies were discharged healthy from Group 2. 6 babies and 3 women died in Group 1 due to acute respiratory distress syndrome. All the three patients had intractable congestive heart failure and severe hypoxia at the time of admission. They came in very sick and died before any definite treatment was started. However, since information was not available for all cases, these factors were not assessed. However, this was in line with a study by Yip who assessed that anemia may not be a direct cause of poor pregnancy outcomes, except in the case of maternal mortality resulting directly from severe anemia due to hypoxia and heart failure [15].

Data available on mean birth weight of 24 and 46 babies was 2056.3 gm and 2616.3 gm in the 2 groups, respectively. Bodeau-Livinec et al. have also found an association between low birth weight and severe anemia [16]. However, due to unavailability of complete data on all outcomes, we did not assess the relation.

Data was available on Complete Blood Count, liver function and kidney function tests on all sixty-six and fifty-nine patients in the 2 groups. The mean differences between Hb, platelet and Bilirubin counts measured between pre- and post-transfusion show significant improvements in both groups. However, since there were more severely anemic women in Group 1, the improvement is clinically significant. A significant mean rise in PCV and hemoglobin was observed with packed cell transfusion (four units with approximately 270 mL per unit) in a study by Philpott et al. [9]. Harrison et al. considered the procedure safer than the exchange transfusion of packed cells in treating severely anemic pregnant women who required emergency surgery or were in advanced labor, since exchange transfusion produced a more rapid rise of PCV [17]. However, our study did not register a significant rise in PCV.

Partial exchange blood transfusion has an undoubted positive impact on the hematological parameters of severely anemic antenatal patient presenting late in pregnancy or in labor (term or preterm) since there were more preterm babies in Group 1 than in Group 2. The mean rank table shows that the improvements were comparable in both groups except for changes in platelet counts which were significantly less in Group 1 showing more improvements in Group 2. A clinical review by Levy and Murphy found that the cause of gestational thrombocytopenia is unclear but poses no risk to either the mother or fetus-neonate [18].

The study suggests that partial exchange blood transfusion and transfusion of packed cell with frusemide cover are comparable modes of transfusion among severely anemic women advanced in pregnancy.

## 6. Limitations

Being a retrospective study, complete records were not found in medical records for some variables, and hence the analysis was restricted for the available data only. A randomized clinical trial is warranted for more efficient results.

## 7. Conclusion

Despite improvement in peripheral health services and all preventive measures, patients of severe anemia at term or in labor are still received. Oral and parenteral irons are slow to act and do not build up hemoglobin to the required levels in a short time before the patients go into labor. The problem is compounded by the fact that iron supplementation during pregnancy is effective in eliminating iron deficiency anemia under controlled and supervised conditions, yet under field conditions, its effectiveness in most countries has been unimpressive [19]. This study was done to validate partial exchange blood transfusion as a method of blood transfusion in antenatal mothers with severe anemia keeping in mind the technical and blood bank limitations that can arise in a small setup. Partial exchange is a procedure that can be done not just at the tertiary level but at primary health level also. The improvements observed in the selected parameters with blood transfusion (with or without exchange) do not suggest that it improves the obstetric outcome too. Research is also needed to clarify the relationship between level of prior anemia, on the one hand, and maternal survival and pregnancy outcome, on the other, to determine the extent to which prophylaxis and treatment modify risk of morbidity and mortality.

## Figures and Tables

**Table 1:** Sociodemographic and reproductive characteristics of anemic pregnant women.

Variable	Category	Group 1	Group 2	*P* value
Age	Continuous	25.6 ± 4	24.7 ± 3.4	0.18
Income	Continuous	1339 ± 1200	1708 ± 2283	0.23
Religion	Hindu	64 (97%)	54 (91.5%)	
	Muslim	2 (3.0%)	5 (8.5%)	0.25
				
Husband education	Illiterate	9 (13.6%)	6 (10.2%)	
	Up to primary	22 (33.3%)	10 (16.9%)	
	Up to middle	12 (18.2%)	16 (27.1%)	
	Secondary and above	23 (34.8%)	27 (45.8%)	0.13
				
Subject's education	Illiterate	24 (36.4%)	17 (28.8%)	
	Up to primary	22 (33.3%)	16 (27.1%)	
	Up to middle	9 (13.6%)	16 (27.1%)	
	Secondary and above	11 (16.7%)	10 (16.9%)	0.29
				
Husband occupation	Unskilled worker	44 (66.7%)	35 (59.3%)	
	Skilled worker	22 (33.3%)	24 (40.7%)	
Subject's occupation	Working	8 (12.1%)	2 (3.4%)	0.10
Diet	Vegetarian	27 (49.1%)	30 (50.8%)	0.85
Obstetrics history				
Parity	1	17 (25.8%)	24 (40.7%)	
	2	16 (24.2%)	21 (35.6%)	
	3-4	33 (50%)	14 (23.7%)	0.01
				
Birth interval	Continuous	27.9 ± 18	29.5 ± 22	0.73

**Table 2:** Nonclinical/medical characteristics of anemic pregnant women.

Variable	Category	Group 1	Group 2	*P* value
Present pregnancy				
Antenatal care	Yes	36 (54.5%)	41 (75.9%)	0.02
Height	Continuous	150.7 ± 4.1	152.2 ± 4.2	0.06
Weight (Kg)	Continuous	51.5 ± 6.6	52.6 ± 6.6	0.36
Antenatal checkup	>3	18 (40%)	24 (58.5%)	0.09
Anemia	Severe	57 (90.5%)	38 (64.4%)	
	Moderate	6 (9.5%)	21 (35.6%)	0.00
Haematinics taken	Yes	25 (52.1%)	31 (93.9%)	0.001
Examination at the time of admission				
General condition	Oriented	58 (92.1%)	56 (98.2%)	
	Disoriented	2 (3.2%)	1 (1.8%)	
	Drowsy	1 (1.6%)	0	
	Other	2 (3.2%)	0	0.38
				
Pulse	Continuous	90 ± 11	88 ± 13	0.33
BP systolic	Continuous	120 ± 18	122 ± 20	0.59
BP diastolic	Continuous	78.8 ± 11.5	78.5 ± 12	0.95
Respiratory rate	Continuous	22.6 ± 15	28.5 ± 9	0.66
Temperature	Afebrile	63 (98.5%)	55 (96.5%)	
	Febrile	1 (1.6%)	2 (3.5%)	0.49
Edema	Yes	36 (56.3%)	18 (31.6%)	0.006
Pallor	Yes	58 (89.2%)	43 (74.1%)	0.03
Icterus	Yes	8 (12.3%)	4 (6.9%)	0.31
Cyanosis	Yes	1 (1.5%)	2 (3.4%)	0.60
Chest	Abnormal	11 (16.9%)	1 (1.8%)	0.005
CVS	Abnormal	12 (19.4%)	2 (3.6%)	0.01
Abdomen	Abnormal	9 (14.7%)	1 (1.9%)	0.01
Fundal height uterus (wks)	Continuous	30.7 ± 4.4	35.9 ± 4.5	0.00
Fetal heart sound	Present	50 (89.3%)	46 (92.0%)	0.65
Uterine contractions	Present	16 (38.1%)	21 (43.8%)	0.58
IUGR	Yes	11 (29.7%)	10 (20.4%)	0.32
Abruption placenta	Yes	3 (6.3%)	1 (1.9%)	0.34
Patient in labour	Yes	16 (32.7%)	24 (50%)	0.08

Figures are in % for categorical variables and in mean ± SD for continuous variables.

**Table 3:** Clinical parameters of baseline complete blood count (CBC), liver and kidney function profile.

Variable	Group 1	Group 2	*P* value
Hb before	5.2 ± 1.5	6.6 ± 2.3	0.00
Hb after	7.2 ± 1.5*	8.6 ± 1.8*	0.00
WBC before	9071 ± 5261	7819 ± 4653	0.18
WBC after	8556 ± 6622	12039 ± 11265*	0.06
Platelet before	1.1 × 10^5^ ± 9 × 10^4^	1.7 × 10^5^ ± 2.8 × 10^5^	0.08
Platelet after	1.0 × 10^5^ ± 8 × 10^4^	2.1 × 10^5^ ± 2.9 × 10^5^*	0.01
ESR before	30.8 ± 15.8	31.5 ± 13.8	0.84
ESR after	30.8 ± 14.3	30.6 ± 18	0.96
MCV before	89 ± 19.7	86.9 ± 22	0.57
MCV after	80 ± 23*	81.9 ± 23	0.75
MCH before	29.6 ± 4.7	31 ± 3.9	0.07
MCH after	30.4 ± 4.3*	31.8 ± 4.7*	0.16
PCV before	19 ± 5.7	21.1 ± 6.6	0.14
PCV after	23.5 ± 5.6*	26 ± 8.0	0.07
* Bilirubin* before	1.3 ± 0.8	0.89 ± 0.6	0.003
* Bilirubin* Post	1.2 ± 0.9	0.79 ± 0.4*	0.004
SGOT before	64 ± 49	49 ± 28	0.08
SGOT after	50.7 ± 29	43.6 ± 30.9	0.26
SGPT before	36.8 ± 26	32.4 ± 19.6	0.37
SGPT after	35 ± 23	31.7 ± 27	0.53
ALP before	349.6 ± 181	337.9 ± 144	0.74
ALP after	298 ± 162*	291 ± 124*	0.81
Blood urea before	34.9 ± 26.9	36.8 ± 35	0.76
Blood urea after	27.6 ± 11*	26.5 ± 8*	0.60
Creatinine before	0.69 ± 0.3	0.67 ± 15	0.66
Creatinine after	0.6 ± 0.2	0.69 ± 0.9	0.57
Sodium before	133 ± 6	134 ± 6	0.73
Sodium after	136 ± 5.7*	135 ± 2.8	0.10
Potassium before	4.4 ± 0.9	4.6 ± 0.6	0.24
Potassium after	4.1 ± 0.5	4.0 ± 0.3*	0.10

*indicates significance within pre- to postcomparison for Group 1 and Group 2 separately.

**Table 4:** Nonparametric analysis of the difference in hematological parameters within and between groups.

Variable	Group 1 (pre- and post-difference) mean rank	Group 2 (pre- and post-difference) mean rank	*P* value
Hb*	2.01 ± 1.6 & 59.3	2.09 ± 1.5 & 59.7	0.09
Platelet	49.3	61.8	0.04
ESR	31.4	40.4	0.06
MCV	42.5	33.9	0.10
MCH	38.2	42.6	0.40
PCV	37.7	35.9	0.73
* Bilirubin*	35.6	40.2	0.35
SGOT	34.4	38.5	0.40
SGPT	36.5	37.5	0.85
Urea	45.6	42.6	0.58
Creatinine	34.2	43.4	0.07
Sodium	30.2	38.1	0.10
Potassium	35.3	33.8	0.76

*Hb is given mean ± sd and mean rank.
